# Coordination of Membrane and Actin Cytoskeleton Dynamics during Filopodia Protrusion

**DOI:** 10.1371/journal.pone.0005678

**Published:** 2009-05-25

**Authors:** Changsong Yang, Matthew Hoelzle, Andrea Disanza, Giorgio Scita, Tatyana Svitkina

**Affiliations:** 1 Department of Biology, University of Pennsylvania, Philadelphia, Pennsylvania, United States of America; 2 The Italian Foundation for Cancer Research (FIRC), Institute for Molecular Oncology and Department of Experimental Oncology, European Institute of Oncology, Milan, Italy; University of Birmingham, United Kingdom

## Abstract

Leading edge protrusion of migrating cells involves tightly coordinated changes in the plasma membrane and actin cytoskeleton. It remains unclear whether polymerizing actin filaments push and deform the membrane, or membrane deformation occurs independently and is subsequently stabilized by actin filaments. To address this question, we employed an ability of the membrane-binding I-BAR domain of IRSp53 to uncouple the membrane and actin dynamics and to induce filopodia in expressing cells. Using time-lapse imaging and electron microscopy of IRSp53-I-BAR-expressing B16F1 melanoma cells, we demonstrate that cells are not able to protrude or maintain durable long extensions without actin filaments in their interior, but I-BAR-dependent membrane deformation can create a small and transient space at filopodial tips that is subsequently filled with actin filaments. Moreover, the expressed I-BAR domain forms a submembranous coat that may structurally support these transient actin-free protrusions until they are further stabilized by the actin cytoskeleton. Actin filaments in the I-BAR-induced filopodia, in contrast to normal filopodia, do not have a uniform length, are less abundant, poorly bundled, and display erratic dynamics. Such unconventional structural organization and dynamics of actin in I-BAR-induced filopodia suggests that a typical bundle of parallel actin filaments is not necessary for generation and mechanical support of the highly asymmetric filopodial geometry. Together, our data suggest that actin filaments may not directly drive the protrusion, but only stabilize the space generated by the membrane deformation; yet, such stabilization is necessary for efficient protrusion.

## Introduction

Leading edge protrusion of migrating cells is the first and essential step of the cell motility cycle, which is mediated by two types of actin-rich protrusive organelles, lamellipodia and filopodia. Despite quite different actin architecture in these two organelles [1], protrusion in both cases involves plasma membrane evagination tightly coordinated with actin filament assembly. A long standing question in the field is which of these two processes plays a primary role. Although actin polymerization is clearly necessary for protrusion, there have been debates about whether growing actin filaments actually push the plasma membrane or they just fill the space created by other membrane-deforming mechanisms and thus stabilize the protrusion [2], [3]. Answering this question has proven to be difficult, probably, because cells normally take special care of synchronizing these two processes.

Most studies over the years focused on the cytoskeletal mechanisms of leading edge protrusion [4], [5]. However, it recently became clear that membrane deformation during protrusion may be regulated by its own mechanisms. Thus, certain membrane-binding and -deforming proteins are involved in protrusion, especially in protrusion of filopodia. These proteins share a conserved I-BAR domain (also known as IMD) [6], [7], which is related to BAR domains of endocytic proteins [8]. Both BAR and I-BAR domains use their arched membrane-binding surfaces to impose and/or stabilize the membrane curvature and induce membrane tubulation. However, the crescent-shaped BAR domains use a concave surface to cause membrane invagination [8], [9], while cigar-shaped I-BAR domains use a convex surface to cause membrane evagination [10], [11]. Furthermore, F-BAR domains (a subtype of BAR domains) assembled on membrane bilayers form a polymeric coat [12] that likely supports the tubular shape of the membrane. I-BARs may be also able to form similar coats as they generate a striated pattern detectable by cryoelectron microscopy when assembled on giant unilamellar vesicles [13].

When expressed in cells, isolated I-BAR domains induce filopodia-like protrusions [6], [10], [11], [14]. Having an extremely asymmetric geometry, filopodia require robust cellular mechanisms to maintain their shape. In conventional filopodia at the leading edge of migrating cells this role is played by a tight internal bundle of long uniformly oriented actin filaments [15]–[17]. Filopodia induction by I-BARs was initially interpreted as a result of their actin-bundling activity [10]. However, subsequent studies did not detect actin-bundling activity of I-BAR in physiological conditions, but only weak and, possibly, non-specific actin-binding activity [11], [18]. Furthermore, the structural organization, and even the presence of actin filaments inside the I-BAR-induced filopodia remain uncertain. Thus, light microscopic data existing on this point provide conflicting information showing either presence [6], [10], [11] or absence [19], [20] of F-actin in I-BAR-induced filopodia, while no electron microscopy (EM) has been done so far with this system.

The anizometric shape of filopodia may also be maintained by polymeric submembranous scaffolds formed by I-BAR domain itself similar to F-BAR domain [12]. Indeed, I-BARs from several proteins can induce tubular invaginations on membrane vesicles in vitro [11], [13]. The scaffolding role of I-BARs is also consistent with the recent analysis of I-BAR-induced filopodia by fluorescence recovery after photobleaching that revealed stable association with the membrane of I-BAR domain from C. elegans, but surprisingly, not of I-BARs from mammalian MIM and IRSp53 proteins, which displayed high dynamics in this assay [13]. Thus, it remains unclear whether I-BAR domain from IRSp53 can form a stable sub-membranous structure and whether membrane deformation alone can drive protrusion of I-BAR-induced filopodia in the cellular context, or the actin cytoskeleton is required for this process, or the reality is somewhere in-between. In any of these outcomes, the structural organization of the actin cytoskeleton and/or potential I-BAR polymeric submembrane assemblies that may provide mechanical support to I-BAR-induced filopodia remain unknown.

In this study, we used a combination of time-lapse fluorescence imaging of living cells and EM to investigate the structure and dynamics of filopodia induced by the I-BAR domain from IRSp53, which is one of the best studied I-BAR-containing proteins playing a key role during leading edge protrusion [21], [22]. Our data show that the I-BAR-dependent membrane deformation may precede actin assembly during filopodia protrusion, but that actin cytoskeleton is required for filopodia elongation and long-term maintenance. Using differential cell extraction in combination with platinum replica EM we revealed the formation of a submembranous I-BAR-containing coat in expressing cells and demonstrated an unusual organization of the actin cytoskeleton in the filopodial interior.

## Results

**Figure 1:**
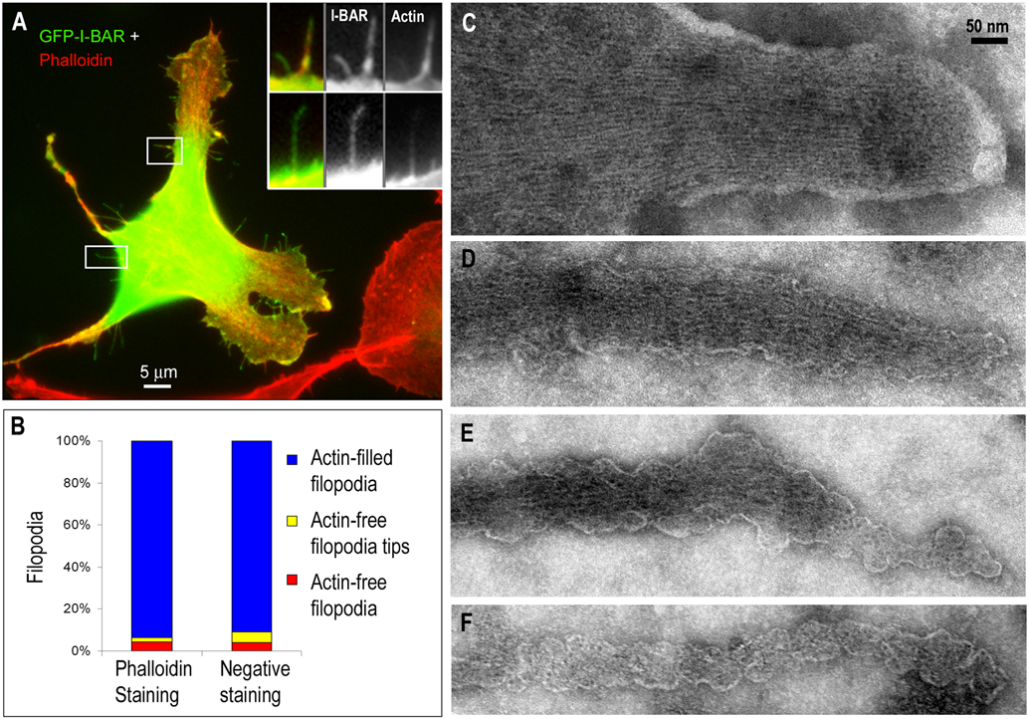
Actin in B16F1 cells expressing GFP-I-BAR from IRSp53. (A) Phalloidin staining. F-actin (red) is present in most, but partially absent from some I-BAR-induced filopodia. GFP-I-BAR is shown in green. Boxed regions are zoomed in insets. Upper inset: F-actin is prominent in the long filopodium and is faint, but still detectable, in the short filopodium. Lower inset: F-actin is not detectable in the distal region of the filopodium. (B) Quantification of F-actin distribution in I-BAR-induced filopodia. Left: Phalloidin-stained filopodia (N = 2080 filopodia from 57 cells). Right: negatively-stained filopodia (N = 154 filopodia from 8 cells). (C–F) Negative staining EM of control and I-BAR-induced filopodia. (C) Control filopodium contains a well-organized bundle of actin filaments extending all the way to the tip. (D–F) Filopodia in cells stably expressing GFP-I-BAR from IRSp53 contain fewer actin filaments, only a fraction (D) or none (E) of which reach the tip. Some rare filopodia do not contain actin filaments (F).

To understand how I-BAR-induced filopodia generate and maintain their shape, we investigated filopodia induced in B16F1 melanoma cells by the GFP-tagged I-BAR domain from IRSp53 (GFP-I-BAR). Over-expression of GFP-I-BAR induced numerous filopodia-like protrusions in B16F1 cells, as previously shown in other cell types [6], [10], [11]. We will subsequently refer to these protrusions as filopodia taking their high length-to-width aspect ratio as a defining feature. Phalloidin staining (Fig. 1A) revealed that truly F-actin-free GFP-I-BAR-induced filopodia were quite rare (4.3±0.5%; mean±SEM) and most filopodia contained F-actin all along the length (92.7±1.0%), although the intensity of phalloidin staining there might be quite low. In some filopodia, however, F-actin could not be detected in distal regions (2.1±0.4%) (Fig. 1B, left).

To test the presence of actin filaments in GFP-I-BAR-induced filopodia with higher precision, we performed negative staining EM, which allows simultaneous visualization of actin filaments and the plasma membrane (Fig. 1C–F). For this analysis, we used a stable GFP- I-BAR-expressing B16F1 cell line. Filopodia of control cells, as expected, contained a tight uniform bundle of actin filaments that spanned the entire length of the filopodium (Fig. 1C). In GFP-I-BAR-expressing cells, most filopodia (90.9%, Fig. 1B, right) also contained actin filaments all along the length; however, filaments were usually less abundant, not as tightly packed, and not all of them extended all the way to the filopodial tip (Fig. 1D). A small fraction (5.2%) of filopodia had distal segments devoid of detectable actin filaments (Fig. 1E). A slightly higher fraction of filopodia in this category as compared to phalloidin stained samples, probably, reflects higher sensitivity of EM. In rare cases (3.9%), filopodia appeared to lack actin filaments completely (Fig. 1F). These findings are consistent with our fluorescence microscopy data and show that F-actin is present in most I-BAR-induced filopodia and absent only in rare cases, preferentially, at the filopodial tips.

**Figure 2:**
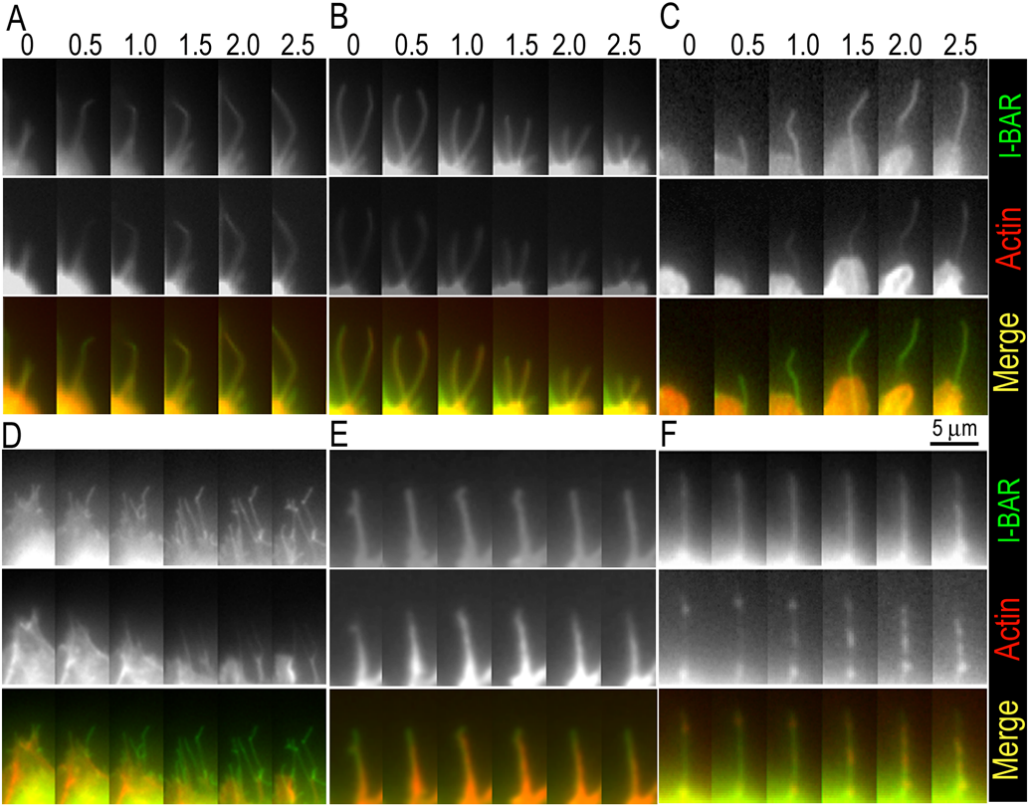
Dynamics of GFP-I-BAR and mCherry- actin in co-expressing cells. (A,B) During protrusion (A) and retraction (B) dynamics of I-BAR (green) and actin (red) are indistinguishable. (C) Actin in a protruding filopodium transiently lags behind the I-BAR, but eventually catches up. (D) Withdrawal of actin produces stationary or retracting actin-free “filopodia”. (E) Actin undergoes polymerization (frames 1–3) and depolymerization (frames 4–6) within a stationary filopodium. (F) Random actin assembly in a stationary actin-free filopodium. The shown categories of filopodia dynamics represent following fractions of I-BAR-induced filopodia: A, 29.1%; B, 40.0%; C, 2.4%; D, 12.1%; E, 2.2%; F, 2.9%, and remaining 11.3% of filopodia showed no dynamics within the period of observation. N = 553 filopodia from 15 cells.

To correlate filopodia behavior with the presence of actin cytoskeleton in their interior, we analyzed the dynamics of GFP-I-BAR and mCherry-actin in double transfected B16F1 cells (Fig. 2). Most of GFP-I-BAR-induced filopodia were stationary, but some were protruding or retracting, similar to the previous report [11]. We found that all protruding and majority of stationary and retracting GFP-I-BAR-positive filopodia contained mCherry-actin. GFP-I-BAR and mCherry-actin often displayed coordinated dynamics during protrusion (Fig. 2A) and retraction (Fig. 2B) of filopodia. However, sometimes actin transiently lagged behind the advancing GFP-I-BAR signal in protruding filopodia (Fig. 2C), suggesting that membrane deformation may precede the actin assembly, albeit in rare cases. Notably, long actin-free segments in GFP-I-BAR-containing filopodia usually formed due to withdrawal of actin (Fig. 2D). Such filopodia commonly remained stationary or retracted. Actin-free filopodial segments might become re-populated with actin filaments, which could polymerize not only at the distal tips of pre-existing actin structures, but in a less organized manner (Fig. 2E,F). These data indicate that the actin-free state of I-BAR-induced filopodia is quite transient and that the protrusive behavior of filopodia strongly correlates with the actin assembly in their interior, even though the mode of assembly may be abnormal.

**Figure 3:**
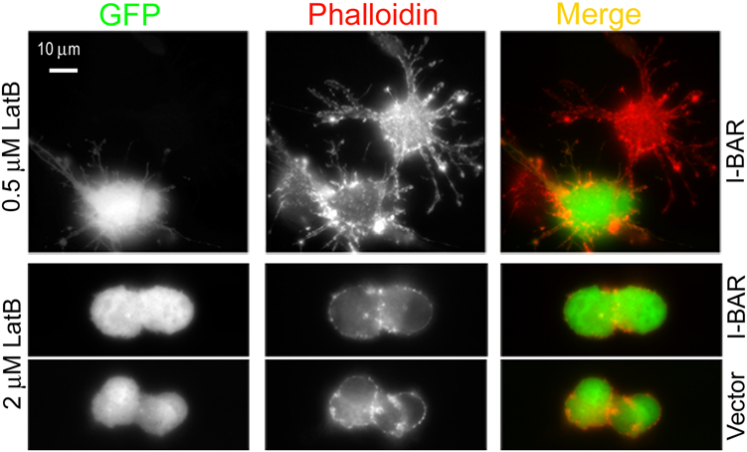
Latrunculin B inhibits I-BAR-induced filopodia. B16F1 cells transfected with GFP-I-BAR or GFP vector (green) were treated with 0.5 µM or 2 µM of LatB for 30 min. F-actin (red) was revealed by phalloidin staining. At low concentration (top row), LatB induces cell arborization, but eliminates the difference in the amount of thin extensions between control and I-BAR-expressing cells. At higher concentration, both GFP-I-BAR-expressing cells (middle row) and GFP-expressing cells (bottom row) completely round up.

To test whether GFP-I-BAR-induced filopodia are stable in the absence of F-actin, we applied an actin-depolymerizing drug, latrunculin B (LatB), to GFP-I-BAR-expressing cells. It was previously reported that low concentrations of actin-depolymerizing drugs do not eliminate I-BAR-induced filopodia [19], but make them less dynamic [11]. After treatment with 0.5 µM LatB, both control and I-BAR-expressing cells acquired an “arborized” shape with multiple branched thin processes filled with remaining actin filaments (Fig. 3, top), microtubules, and intermediate filaments (not shown), as typical for actin-depolymerizing drugs. However, there was no obvious difference between control and GFP-I-BAR-expressing cells in the amount of linear protrusions. Furthermore, a higher LatB concentration (2 µM) caused complete retraction of all protrusions and rounding of both control and GFP-I-BAR-expressing cells (Fig. 3, bottom), suggesting that GFP-I-BAR-induced filopodia require actin cytoskeleton for long-term maintenance.

Next, we investigated the structural organization of GFP-I-BAR-induced filopodia by platinum replica EM, which is particularly useful for analyses of the cytoskeleton in detergent-extracted samples [23], [24]. To unambiguously identify expressing cells, we used the stable I-BAR-expressing cell line or correlative microscopy, in which the same cell is first observed by fluorescence light microscopy and then by replica EM [23], [24].

**Figure 4:**
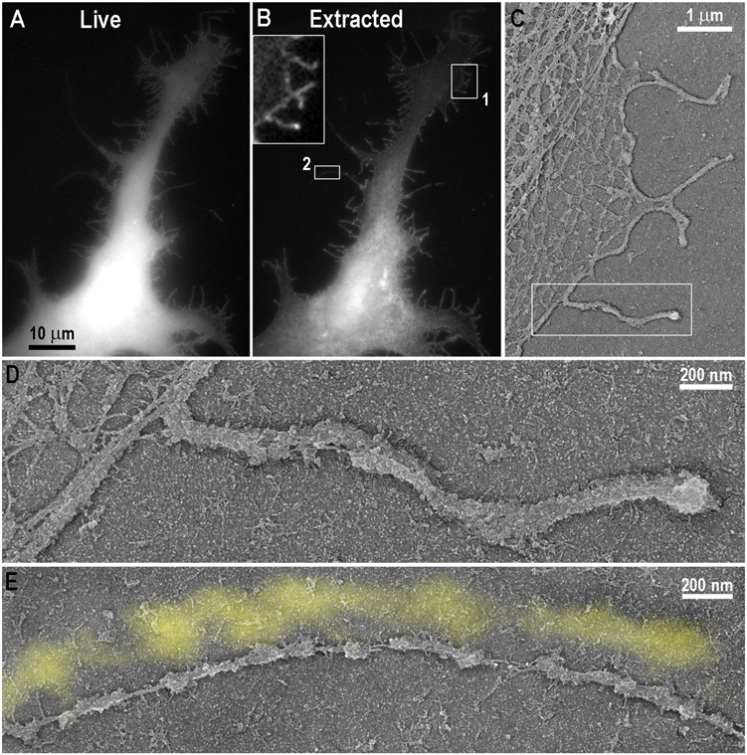
I-BAR-IRSp53 forms a submembranous coat. Correlative fluorescence and platinum replica EM of GFP-I-BAR expressing cells. (A,B) Expressing cell in living state (A) and after mild detergent extraction in the presence of PEG (B). Box 1 is enlarged in inset. (C–E) Non-extracted I-BAR fluorescence corresponds to coating material associated with filopodia and cell edges. (C) Correlative EM of box 1 from B. (D) Enlarged box from C. (E) Correlative EM of box 2 from B. Actin filaments are visible through gaps in the coat (arrows). Yellow shade in E represents the GFP-I-BAR image of the same region zoomed to the scale.

When cells were extracted in the presence of polyetheleneglycol (PEG), which helps to preserve weakly bound cytoskeletal components [23], [24], significant amount of GFP-I-BAR fluorescence remained associated with the cytoskeleton of expressing cells, especially in filopodia and at the cell edges (Fig. 4A, B). In the corresponding replica EM images, filopodia and cell edges were coated with finely structured material, while actin filaments, sometimes as few as one, could be seen through gaps in the coating (Fig. 4C–E). Correlative EM showed that this material corresponded to the remaining I-BAR fluorescence in extracted cells (Fig. 4E). These data suggest that the GFP-I-BAR domain, alone or together with other cellular proteins, forms multimolecular complexes under the membrane.

**Figure 5:**
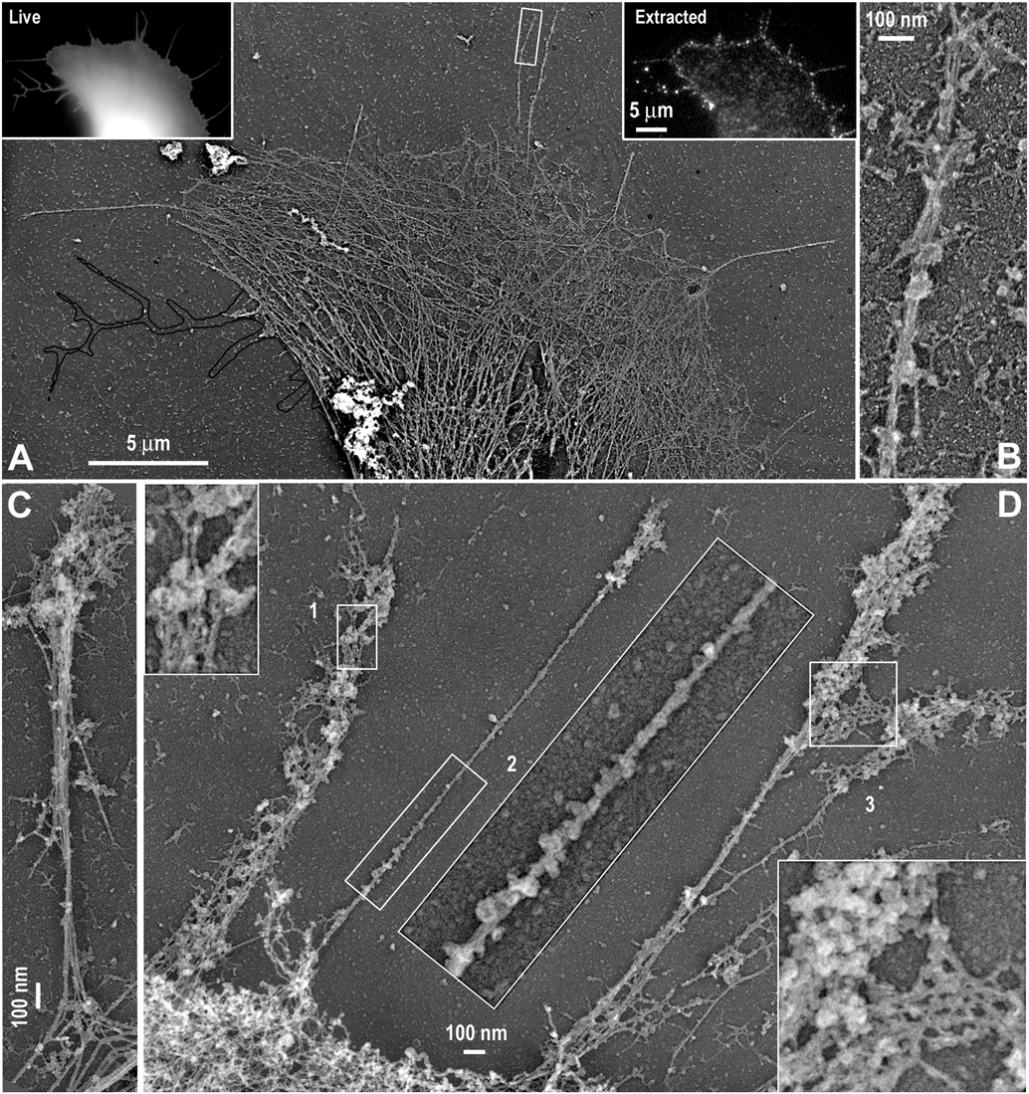
Organization of the cytoskeleton in I-BAR-induced filopodia. (A) Correlative platinum replica EM of a GFP-I-BAR-expressing cell in living state (left inset) and after stringent detergent extraction in the absence of PEG (right inset). Actin cytoskeleton is present in most I-BAR-positive filopodia except for those marked by the black outline projected from the live fluorescence image. (B) Zoomed boxed region from A shows aberrant actin filament organization in the I-BAR-induced filopodium. (C,D) Replica EM of cells stably expressing GFP-I-BAR shows variable number of actin filaments inside I-BAR-induced filopodia and frequent association of filopodia with reticular material. Boxes 1–3 are enlarged in adjacent panels.

When I-BAR-expressing cells were extracted in more stringent conditions in the absence of PEG (Fig. 5), the GFP-I-BAR fluorescence (Fig. 5A, insets) and corresponding submembranous material were largely removed exposing the structure of the underlying cytoskeleton (Fig. 5). Correlative replica EM showed that the majority of GFP-I-BAR-positive filopodia contained actin filaments in their interior (91.3%, N = 418 filopodia from 7 cells) and only few filopodia (8.7%) were actin-free (Fig. 5A). As compared to normal filopodia, the GFP-I-BAR-induced filopodia contained fewer actin filaments, sometimes only one or two (Fig. 5D). Furthermore, filopodial filaments were frequently poorly bundled and did not span the entire length of the filopodium, but could begin and end at random places within the filopodium (Fig. 5B, C). These findings are consistent with our time-lapse imaging experiments (see Fig. 2F) and suggest that actin assembly in GFP-I-BAR-induced filopodia is poorly regulated and not limited to filopodia tips, like in normal filopodia [25]. Additionally, we found prominent patches of granular and reticular material associated with tips and/or sides of filopodia in GFP-I-BAR-expressing cells (Fig. 5C, D). This material is likely homologous to the I-BAR-containing coat revealed in samples extracted in the presence of PEG and its changed morphology may be due to different extraction conditions.

**Figure 6:**
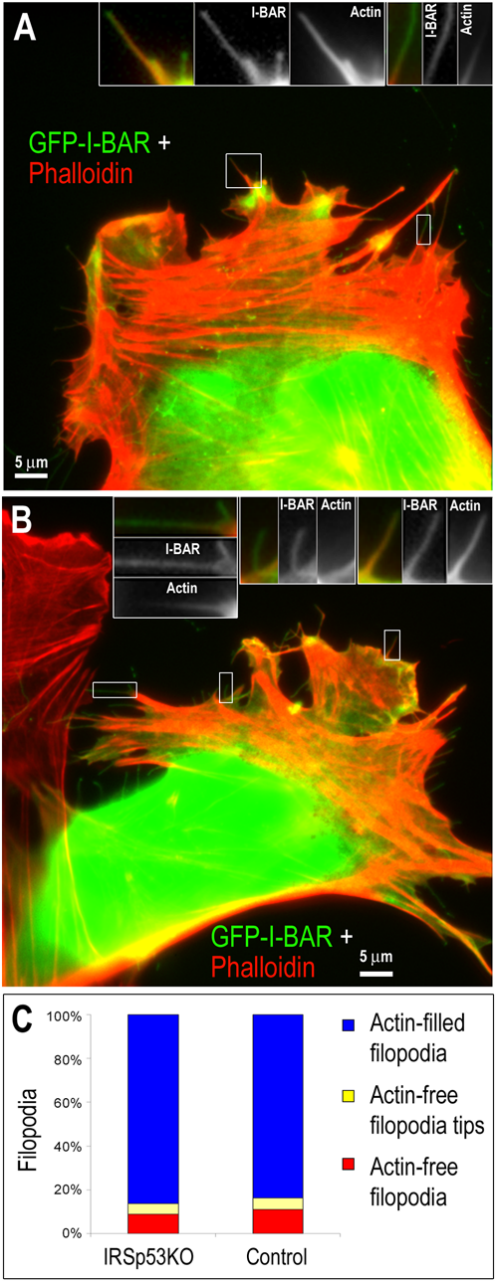
F-Actin in IRSp53 knockout and control MEFs expressing GFP-I-BAR from IRSp53. Phalloidin staining of IRSp53 knockout (A) or control (B) cells. F-actin (red) is present in most, but partially absent from some I-BAR-induced filopodia. GFP-I-BAR is shown in green. Boxed regions are zoomed in insets. (A) Left inset: F-actin is present throughout the long filopodium, but not detectable in the short filopodium. Right inset: F-actin is not detectable in the distal region of the filopodium. (B) Left inset: F-actin is not detectable in the distal region of the filopodium. Middle inset: F-actin is not detectable in the filopodium. Right inset: F-actin is present throughout filopodium (C) Quantification of F-actin distribution in I-BAR-induced filopodia in IRSp53 knockout (left) and control (right) MEFs. N = 678 filopodia from 33 IRSp53 knockout cells and 649 filopodia from 35 control cells.

The frequent presence of actin filaments in I-BAR-induced filopodia raises a possibility of their active recruitment into these structures. Indeed, the I-BAR domain of IRSp53 can dimerize [19], while the C-terminal domains of IRSp53 are able to interact with a number of actin-binding proteins [21]. Therefore, one can imagine that ectopically expressed I-BAR domain dimerizes with the endogenous full length IRSp53, which then recruits actin into induced filopodia through its interaction partners. To address this possibility, we used mouse embryo fibroblasts (MEFs) from IRSp53 knockout mice [26]. Over-expression of GFP-I-BAR induced filopodia both in IRSp53 knockout MEFs and in control cells stably re-expressing IRSp53 (Fig. 6). Most filopodia in both cell lines contained F-actin (86.4±1.0% in IRSp53 knockout MEFs and 83.7±0.9% in control cells; N = 649 filopodia from 35 control cells and 678 filopodia from 33 IRSp53 knockout cells), indicating that the frequency of actin appearance in filopodia does not depend on the presence of full-length IRSp53 in cells. A slightly higher fraction of actin-free filopodia in MEFs than in B16F1 cells may reflect cell type-specific differences.

## Discussion

A tight parallel bundle of long continuous actin filaments is believed to be necessary for mechanical stability and protrusion of filopodia and it is generally considered as a hallmark of highly elongated membrane protrusions [1]. In contrast to this notion, the membrane binding and bending I-BAR domain of IRSp53 has been proposed to induce filopodia-like protrusions in the absence of actin filaments [11], [20]. These intriguing findings raised a question about structural elements that might be responsible for generation and maintenance of these highly asymmetric shapes. To address this question, we performed detailed dynamic and structural analyses of filopodia induced by IRSp53 I-BAR in B16F1 cells.

Our data show that actin filaments are not only present in the majority of I-BAR-induced filopodia, but they are essential for their protrusion and long-term maintenance. Surprisingly, our data reveal that in order to support the shape and protrusive behavior of filopodia, actin cytoskeleton may not be as perfectly organized as in normal filopodia, but may consist of few relatively short and poorly aligned filaments undergoing random polymerization. Low abundance of actin filaments in many I-BAR-induced filopodia makes them poorly detectable by fluorescence microscopy and may explain why actin-free filopodia appeared more abundant in other studies [19], [20]. Another possibility is that different cells types vary in their ability to maintain actin-free filopodia, as we found for MEFs versus B16F1 cells.

Our findings suggest that a typical bundle of parallel actin filaments is not necessary for generation and mechanical support of the highly asymmetric filopodial geometry, but unconventionally organized actin filaments are able to produce and maintain filopodia. Although this unusual actin organization is found in conditions of over-expression of a constitutively active protein, we also found recently an unusual network-like organization of actin filaments in the naturally occurring dendritic filopodia in cultured hippocampal neurons (unpublished data). These data suggest that the cytoskeletal organization of morphologically similar filopodia may vary significantly depending on physiological and/or experimental conditions, thus changing the existing paradigm of filopodia protrusion.

These findings also raise a semantic question of whether “filopodia” is an appropriate term for these structures, as it is currently believed that filopodia must contain uniformly oriented and tightly bundled actin filaments elongating at the distal barbed ends, as in the best studied leading edge filopodia of migrating cells. However, the term filopodia was initially introduced to define various surface protrusions sharing only one feature, a high length-to-width ratio, independent on their internal organization. Since very few filopodia types have been analyzed systematically, it remains unknown to which extent the specific features of the leading edge filopodia apply to all thin elongated protrusions. We propose to use “filopodia” as a generic term to designate any highly asymmetric spike-like membrane protrusions, while specific types of filopodia can be indicated by relevant adjectives, such as I-BAR-induced filopodia in our case.

Despite the frequent presence and an essential role of actin filaments in I-BAR-induced filopodia, we confirm here the previous data [19], [20] that the actin-free filopodia also exist in these cells, albeit at low frequency. In addition to fluorescence microscopy, we used two different EM techniques to validate this point and rule out potential sensitivity problems. Importantly, the majority of actin-free filopodia in our experiments were produced not by extension of an “empty” membrane tube, but by actin withdrawal from the pre-existing actin-positive filopodia. This finding underscores again the importance of the actin cytoskeleton and the relative weakness of I-BAR in generating filopodia in vivo. However, it has recently been shown that the purified I-BAR domain can induce tubulation of PIP2-containing lipid vesicles in vitro [11], [13]. This apparent conflict with our data is likely explained by very high concentrations of PIP2 and I-BAR used in this reconstitution system, which were much above those existing in cells. Yet, we were able to detect rare events of membrane extension preceding actin assembly during filopodia protrusion, suggesting that conditions similar to those used in the reconstitution system may sometimes form in vivo. Although very rare, such events are very important to demonstrate the ability of I-BAR domain to drive membrane protrusion in vivo in the apparent absence of the cytoskeleton.

Extreme deformations of plasma membrane, like those in filopodia, are energetically unfavorable and therefore require mechanical support, which is normally provided by the cytoskeleton. However, other membrane-associated proteins also can modulate the shape of the membrane, usually by forming polymeric scaffolds [12], [27]. We showed here that a similar mechanism seems to function in actin-free I-BAR-induced filopodia, as I-BAR domain forms a submembranous coat in filopodia and along cell edges. The coat is clearly exaggerated in these over-expressing conditions as compared to normal cells. However, since full-length IRSp53 is enriched at filopodial tips [28], it is possible that a similar I-BAR-dependent scaffold represents a part of the filopodial tip complex in normal conditions [17] and functions there to generate initial membrane deformation that promotes polymerization of actin filaments into the created space.

In the endogenous IRSp53 protein, the I-BAR domain exists in the context of other domains, which are able to recruits several regulators of actin polymerization [21], [22]. Some of these interacting proteins, such as small GTPases Cdc42 [29] and Rac1 [30], likely activate IRSp53 and expose the I-BAR domain, which then induces the membrane curvature at specific sites on the plasma membrane. Others, such as activators of the Arp2/3 complex WAVE2 [30] and N-WASP [20] and barbed end-binding proteins Mena [29], Eps8 [31], and mDia1 [32], may induce actin polymerization specifically at these sites of membrane evagination and also to define the type of actin filament organization within induced protrusions. These properties of IRSp53 may explain why uncoupling between membrane deformation and actin assembly is virtually undetectable in normal conditions.

**Figure 7:**
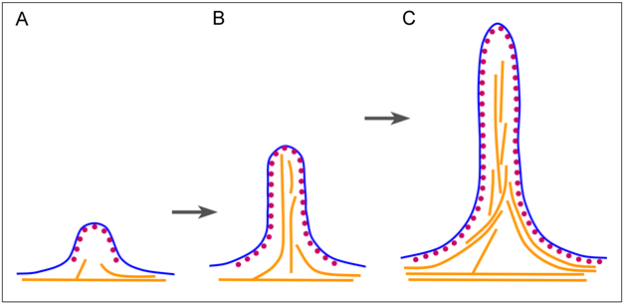
Model for formation of I-BAR-induced filopodia. (A) I-BAR (purple dots) generates and partially supports the initial plasma membrane (blue line) deformation, possibly, through formation of polymeric structures. (B) Actin filaments (orange lines) fill the space generated by I-BAR through stochastic polymerization. (C) Filopodia protrusion occurs through transient formation of empty space at the filopodial tips, which may be very small, followed by actin assembly into this space to stabilize the protrusion.

In summary, the prominent ability of the isolated I-BAR domain to deform the membrane and thus to uncouple actin and membrane machineries allowed us to determine the relative contribution of the membrane deformation and the actin assembly to filopodia protrusion (Fig. 7). We propose that actin filaments in these conditions may not directly drive protrusion, but only provide mechanical support to the membrane deformation, which is induced and transiently stabilized by polymeric assemblies of the GFP-I-BAR domain of IRSp53. Yet, actin cytoskeleton is necessary for long-term stabilization and efficient protrusion of filopodia. Notably, the modes of actin filament organization and assembly may be quite different from a conventional actin filament bundle, but still able to support the highly asymmetric geometry and protrusive behavior of GFP-I-BAR-induced filopodia. These findings significantly contribute to our understanding of the cross-talk between the membrane and the actin cytoskeleton during leading edge protrusion of migrating cells.

## Materials and Methods

### Reagents

The GFP-IRSp53-I-BAR construct has been described [31]. The mCherry-actin construct was cloned from pEGFP-actin into mCherry-C1 vector (Clontech). LatB was from Calbiochem (La Jolla, CA), Alexa Fluor 594-labeled phalloidin was from Invitrogen (Carlsbad, CA), and other reagents were from Sigma (St. Louis, MO).

### Cell culture and microscopy

B16F1 mouse melanoma cells were cultured as described [33]. Transient transfection was performed using Lipofectamine 2000 (Invitrogen). Stable cell line expressing GFP-IRSp53-I-BAR was established by FACS and G418 selection. IRSp53 knockout cells were spontaneously immortalized cells from IRSp53 knockout mouse embryos infected either with pBABE-puro or pBABE-puro-IRSp53 [26]. MEFs were cultured in DMEM-Glutamax-1 medium supplemented with 20% FBS, 1× Pen-Strep, and 1 µg/ml puromycin. Light microscopy was performed using Eclipse TE2000-U inverted microscope (Nikon) equipped with Planapo 100×1.3 NA objective and Cascade 512B CCD camera (Photometrics) driven by Metamorph imaging software (Molecular Devices). For live-cell imaging, cells were transferred into phenol red–free L-15 medium supplemented with 10% FBS and kept on the microscope stage at 35°C during observation. To detect F-actin in filopodia of GFP-I-BAR expressing cells, cells were fixed with 4% paraformaldehyde in PBS, permeabilized by 1% Triton X-100 in PBS for 5 min, and stained with Alexa Fluor-594 phalloidin. For quantification purposes, filopodia with F-actin-free tips were defined as filopodia lacking detectable phalloidin signal within a distance of 0.5 µm or more from the tip of GFP-I-BAR signal in the same filopodium. Statistical analysis of filopodia dynamics was performed using 5 min-long dual-color (GFP-I-BAR and mCherry-actin) time-lapse sequences acquired with 15-s intervals between frames.

For negative staining EM, cells were treated with 0.01% saponin in PEM buffer (100 mM PIPES-KOH, pH 6.9, 1 mM MgCl_2_, 1 mM EGTA) containing 2 µM phalloidin for 10 min at 4°C, fixed with 1% glutaraldehyde in 0.1 M Na-cacodylate (pH 7.3) at room temperature, and stained with 2% sodium silicotungstate (pH 7) containing 0.1% trehalose [34]. For quantification purposes, filopodia with F-actin-free tips were defined as filopodia lacking detectable actin filaments within a distance of 0.2 µm or more from the tip of the same filopodium. Cells for platinum replica EM were processed as described [23], [24]. Briefly, cells were extracted for 5 min at room temperature with 1% Triton X-100 in PEM buffer containing 2 µM phalloidin with or without 2% PEG (MW 35,000), followed by fixation with 2% glutaraldehyde in 0.1 M Na-cacodylate (pH 7.3). Correlative EM was performed as described using cells growing on marked coverslips [23], [24]. Quantification of F-actin positive or F-actin-free filopodia was done using correlative EM of I-BAR-expressing cells extracted without PEG. Samples were analyzed using JEM 1011 transmission electron microscope (JEOL USA) operated at 100 kV. Images were captured by ORIUS 835.10W CCD camera (Gatan) and presented in inverted contrast.

## References

[1] Chhabra ES, Higgs HN (2007). The many faces of actin: matching assembly factors with cellular structures.. Nat Cell Biol.

[2] Bereiter-Hahn J (2005). Mechanics of crawling cells.. Med Eng Phys.

[3] Loitto VM, Forslund T, Sundqvist T, Magnusson KE, Gustafsson M (2002). Neutrophil leukocyte motility requires directed water influx.. J Leukoc Biol.

[4] Pollard TD, Borisy GG (2003). Cellular motility driven by assembly and disassembly of actin filaments.. Cell.

[5] Le Clainche C, Carlier MF (2008). Regulation of actin assembly associated with protrusion and adhesion in cell migration.. Physiol Rev.

[6] Yamagishi A, Masuda M, Ohki T, Onishi H, Mochizuki N (2004). A novel actin bundling/filopodium-forming domain conserved in Insulin receptor tyrosine kinase substrate p53 and Missing in metastasis protein.. J Biol Chem.

[7] Millard TH, Dawson J, Machesky LM (2007). Characterisation of IRTKS, a novel IRSp53/MIM family actin regulator with distinct filament bundling properties.. J Cell Sci.

[8] Dawson JC, Legg JA, Machesky LM (2006). Bar domain proteins: a role in tubulation, scission and actin assembly in clathrin-mediated endocytosis.. Trends Cell Biol.

[9] Itoh T, De Camilli P (2006). BAR, F-BAR (EFC) and ENTH/ANTH domains in the regulation of membrane-cytosol interfaces and membrane curvature.. Biochim Biophys Acta.

[10] Millard TH, Bompard G, Heung MY, Dafforn TR, Scott DJ (2005). Structural basis of filopodia formation induced by the IRSp53/MIM homology domain of human IRSp53.. Embo J.

[11] Mattila PK, Pykalainen A, Saarikangas J, Paavilainen VO, Vihinen H (2007). Missing-in-metastasis and IRSp53 deform PI(4,5)P2-rich membranes by an inverse BAR domain-like mechanism.. J Cell Biol.

[12] Frost A, Perera R, Roux A, Spasov K, Destaing O (2008). Structural basis of membrane invagination by F-BAR domains.. Cell.

[13] Saarikangas J, Zhao H, Pykalainen A, Laurinmaki P, Mattila PK (2009). Molecular mechanisms of membrane deformation by I-BAR domain proteins.. Curr Biol.

[14] Saarikangas J, Hakanen J, Mattila PK, Grumet M, Salminen M (2008). ABBA regulates plasma-membrane and actin dynamics to promote radial glia extension.. J Cell Sci.

[15] Small JV (1988). The actin cytoskeleton.. Electron Microsc Rev.

[16] Lewis AK, Bridgman PC (1992). Nerve growth cone lamellipodia contain two populations of actin filaments that differ in organization and polarity.. J Cell Biol.

[17] Svitkina TM, Bulanova EA, Chaga OY, Vignjevic DM, Kojima S (2003). Mechanism of filopodia initiation by reorganization of a dendritic network.. J Cell Biol.

[18] Lee SH, Kerff F, Chereau D, Ferron F, Klug A (2007). Structural basis for the actin-binding function of missing-in-metastasis.. Structure.

[19] Suetsugu S, Murayama K, Sakamoto A, Hanawa-Suetsugu K, Seto A (2006). The RAC binding domain/IRSp53-MIM homology domain of IRSp53 induces RAC-dependent membrane deformation.. J Biol Chem.

[20] Lim KB, Bu W, Goh WI, Koh E, Ong SH (2008). The Cdc42 effector IRSp53 generates filopodia by coupling membrane protrusion with actin dynamics.. J Biol Chem.

[21] Scita G, Confalonieri S, Lappalainen P, Suetsugu S (2008). IRSp53: crossing the road of membrane and actin dynamics in the formation of membrane protrusions.. Trends Cell Biol.

[22] Mattila PK, Lappalainen P (2008). Filopodia: molecular architecture and cellular functions.. Nat Rev Mol Cell Biol.

[23] Svitkina TM, Borisy GG, Celis J (2006). Correlative light and electron microscopy studies of cytoskeletal dynamics.. Cell Biology: A Laboratory Handbook; 3rd Edition.

[24] Svitkina T (2007). Electron microscopic analysis of the leading edge in migrating cells.. Methods Cell Biol.

[25] Mallavarapu A, Mitchison T (1999). Regulated actin cytoskeleton assembly at filopodium tips controls their extension and retraction.. J Cell Biol.

[26] Weiss SM, Ladwein M, Schmidt D, Ehinger J, Lommel S (2009). IRSp53 links the enterohemorrhagic E. coli effectors Tir and EspFU for actin pedestal formation.. Cell Host Microbe.

[27] Doherty GJ, McMahon HT (2008). Mediation, modulation, and consequences of membrane-cytoskeleton interactions.. Annu Rev Biophys.

[28] Nakagawa H, Miki H, Nozumi M, Takenawa T, Miyamoto S (2003). IRSp53 is colocalised with WAVE2 at the tips of protruding lamellipodia and filopodia independently of Mena.. J Cell Sci.

[29] Krugmann S, Jordens I, Gevaert K, Driessens M, Vandekerckhove J (2001). Cdc42 induces filopodia by promoting the formation of an IRSp53:Mena complex.. Curr Biol.

[30] Miki H, Yamaguchi H, Suetsugu S, Takenawa T (2000). IRSp53 is an essential intermediate between Rac and WAVE in the regulation of membrane ruffling.. Nature.

[31] Disanza A, Mantoani S, Hertzog M, Gerboth S, Frittoli E (2006). Regulation of cell shape by Cdc42 is mediated by the synergic actin-bundling activity of the Eps8-IRSp53 complex.. Nat Cell Biol.

[32] Fujiwara T, Mammoto A, Kim Y, Takai Y (2000). Rho small G-protein-dependent binding of mDia to an Src homology 3 domain-containing IRSp53/BAIAP2.. Biochem Biophys Res Commun.

[33] Yang C, Czech L, Gerboth S, Kojima S, Scita G (2007). Novel roles of formin mDia2 in lamellipodia and filopodia formation in motile cells.. PLoS Biol.

[34] Small J, Rottner K, Hahne P, Anderson KI (1999). Visualising the actin cytoskeleton.. Microsc Res Tech.

